# Cerebrovascular accident in a child with precursor B-cell acute lymphoblastic leukemia and coronavirus disease 2019: a case report

**DOI:** 10.1186/s13256-022-03672-5

**Published:** 2022-12-06

**Authors:** Hanie Karimi, Roham Sarmadian, Abolfazl Gilani, Poorya salajegheh, Habibe Nejad Biglari, Mahsa Gholizadeh

**Affiliations:** 1 Alumna of Medicine, Tehran University of Medical Sciences, Tehran, Iran; 2 Department of Infectious Disease, Arak University of Medical Sciences, Arak, Iran; 3 Department of Pediatric Surgery, Tehran University of Medical Sciences, Tehran, Iran; 4 Department of Pediatric Oncology, Kerman University of Medical Sciences, Kerman, Iran; 5 Department of Pediatric Neurology, Kerman University of Medical Sciences, Kerman, Iran

**Keywords:** COVID-19, SARS-CoV-2, Pre-B-cell leukemia, Cerebrovascular accident, Brain ischemia

## Abstract

**Background:**

Coronavirus disease 2019 can lead to rare but severe and life-threatening diseases in susceptible high-risk populations, including patients with immunodeficiency. A rare event in this report is stroke following COVID-19 disease in a patient with an immunocompromised background due to leukemia and anti-cancer treatments.

**Case presentation:**

A 6-year-old iranian girl with precursor B-cell leukemia receiving vincristine therapy presented with fever and absolute neutrophil count < 500. Her severe acute respiratory syndrome coronavirus 2 polymerase chain reaction test was positive. During hospitalization, she had abrupt onset tachypnea, reduced O_2_ saturation, and generalized tonic–clonic seizures treated with phenytoin and levetiracetam. Right parietal lobe ischemia was found on a brain computed tomography scan, and the cerebrospinal fluid polymerase chain reaction test was positive for severe acute respiratory syndrome coronavirus 2. Several days later, she developed lower extremity paralysis and speech impairment, so speech therapy and physiotherapy were initiated. The patient also received dexamethasone, mannitol, heparin, and remdesivir. She was discharged with enoxaparin and levetiracetam. Chemotherapy resumed 2 weeks following discharge. Her speech and walking improved after 10 months of follow-up, and bone marrow aspiration showed total remission.

**Conclusion:**

Owing to the link between coronavirus disease 2019 and hematologic cancers with hypercoagulopathy and the tendency of patients with leukemia to have coronavirus disease 2019 complications, children with leukemia as well as suspected coronavirus disease 2019 must be hospitalized to prevent blood clot formation.

## Introduction

The coronavirus disease 2019 (COVID-19), first reported in Wuhan, China, was caused by severe acute respiratory syndrome coronavirus (SARS-CoV-2). It then became a pandemic, leading to high morbidity and mortality worldwide [1–3]. Although all ages can be affected, COVID-19 has a lower clinical impact, milder course, and better clinical outcomes in children than in adults [4]. Common symptoms of COVID-19 are fever, cough, and dyspnea. In addition, it can affect other organs, resulting in hematological, neurological, cardiac, and renal complications [5, 6]. Owing to the detection of SARS-CoV-2 in cerebrospinal fluid (CSF) and brain tissue during the autopsy, several studies have suggested that the virus may be neuroinvasive [7–9]. The neurological symptoms include encephalitis, encephalopathy, Guillain–Barré syndrome, epileptic seizures, and stroke [10]. In a retrospective study on 214 patients in Wuhan, China, neurological complications were observed in 36.4% of patients with COVID-19 [11]. During the COVID-19 pandemic, the incidence of ischemic stroke has been approximately 5%. Patients infected with COVID-19 who experience a stroke are more likely to be elderly and have hypertension [12]. Despite annual incidence estimates of 1.3–1.72 instances per 100,000 children in Europe and North America, little is known regarding the occurrence of ischemic stroke in pediatric COVID-19 [13, 14].

Immunocompromised populations, such as patients with hematological cancers, appear to be at higher risk of severe COVID-19-related illnesses, such as cerebrovascular disorders, than the general population [15–17]. Moreover, children with acute lymphoblastic leukemia were found to have the highest risk of both ischemic stroke and intracerebral hemorrhage [18, 19]. Therefore, COVID-19 infection raises the risk of stroke in children with acute lymphoblastic leukemia (ALL) owing to their immunodeficiency and hypercoagulability. We present the case of a 6-year-old girl with pre-B-cell ALL who was hospitalized with the diagnosis of COVID-19 and experienced a cerebrovascular accident during hospitalization.

## Case presentation

A 6-year-old iranian girl with a history of pre-B-cell acute lymphoblastic leukemia (pre-B-cell ALL) was admitted to the hospital. Her leukemia was identified 8 months prior to her hospitalization with the 46XX blast cell karyotype, positive for terminal deoxynucleotidyl transferase (TdT), CD10, CD34, CD19, Human Leukocyte Antigen-DR isotype (HLA-DR), and cytoplasmic immunoglobulin M (CyIgM), placing her in the standard-risk group. The patient was at the beginning of the maintenance treatment, which included vincristine injections and examinations every 35 days. Her family resided in a rural area and had a lower-middle-class income. Family medical history had no evidence of any cancerous or hematologic diseases. Patient was not exposed to any dangerous factors (radiation and chemicals) during her life or fetal period. She was the firstborn child and the mother’s first pregnancy. Her weight at birth was 3000 g. Mother’s age at birth was 20 years of age and the father’s was 27 years of age.

**Fig. 1:**
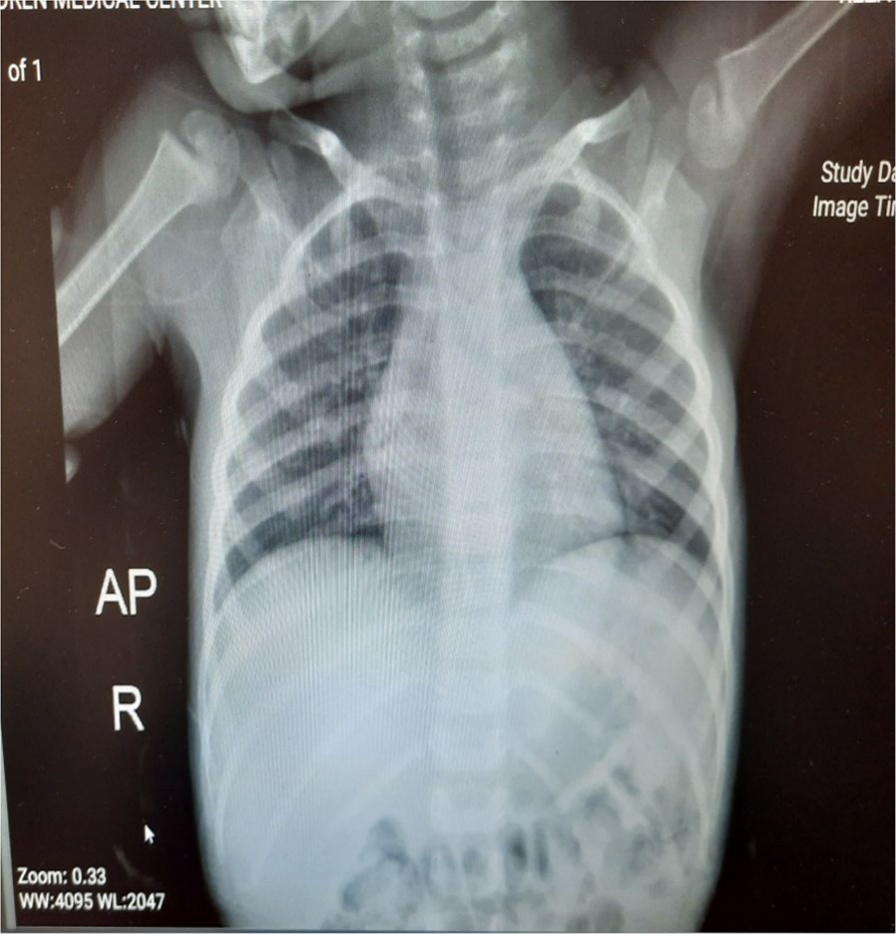
Chest X-ray taken on the second day of hospitalization, showing normal field of both lungs

Three days before admission, the child experienced a rapid onset of fever with no other symptoms. With the onset of fever, the patient received supportive treatment and acetaminophen syrup 6 cc/kg/8 h, which had no effect on improving the patient’s fever. At the beginning of admission the hospital, vital signs were: temperature (T) 39.8 °C, pulse rate (PR) 86 beats per minute, respiratory rate (RR) 13 breaths per minute, blood pressure (BP) 100/70 mmHg, and O2 saturation 97%. Physical examination revealed pale skin and splenomegaly. Lymphadenopathy, hepatomegaly, and the presence of a central nervous system (CNS) disorder/leukemia were not observed. The neurological examination, including all cranial and peripheral nerve examinations, was normal. She was admitted for additional examination, and the initiation of antibiotic treatment after a laboratory test revealed that her absolute neutrophil count (ANC) was lower than 500/µl. She was treated empirically with intravenous vancomycin 500 mg and meropenem 500 mg three times daily. The blood culture was negative after 48 h with a normal chest X-ray (Fig. 1) The patient’s laboratory test results were as follows: red blood cell count (RBC) 4600, white blood cell count (WBC) 1700/µl, ANC 1100/µl, Hemoglobin 9.3 gr/dl, Platelets 47,000/µl, PT 16s, International normalized ratio 1.1, Prothrombin time test 24s, Alanine transaminase 12, Aspartate aminotransferase 14, Alkaline phosphatase 20, Blood Urea Nitrogen 11, Creatinine 0.8, Erythrocyte sedimentation rate 35, C-Reactive Protein 1+; urine analysis: WBC 2–3, RBC 0; epithelial cell: 1–2; crystals: not seen; cast: not seen; bacteria: not seen; and the other test results were normal.

On the third day of hospitalization, which corresponded to the sixth day of the onset of fever, the patient developed sudden-onset tachypnea and lowered oxygen saturation to 88% in ambient air, which increased to 94% when using a reservoir oxygen mask. After partial stabilization, the patient’s oxygen saturation reached 90%, and generalized tonic–clonic seizures commenced concurrently. Despite receiving IV midazolam 5 mg twice, the seizure persisted.

**Fig. 2:**
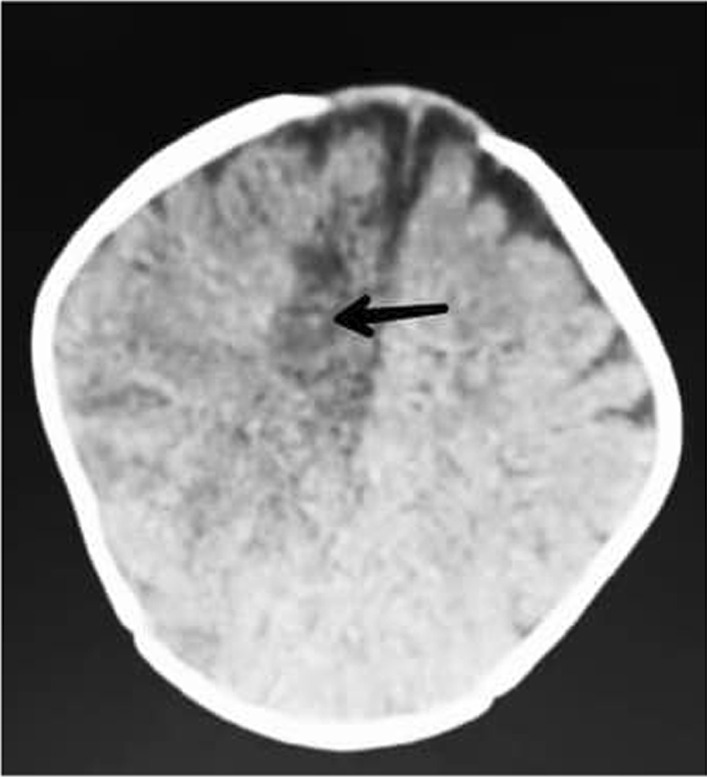
Brain computed tomography scan showing right parietal lobe ischemia (black arrow)

The patient was immediately transferred to the pediatric intensive care unit (PICU) and was intubated. According to the patient’s symptoms, a cerebrovascular accident (CVA) was suspected; therefore, she underwent a brain computed tomography (CT) scan without contrast, which revealed an ischemic CVA in the right parietal lobe, resulting in edema and midline shift of the brain. (Fig. 2) Therefore, treatment procedures, including intravenous dexamethasone 4 mg daily and mannitol 12 g, were started for the patient.

The patient’s seizure was controlled by administering intravenous phenytoin 500 mg and levetiracetam 1 g. Owing to the patient’s low platelet count, heparin was administered at a therapeutic dose of 1800 units to prevent thrombosis development, and the infusion was continued at a dose of 700 units per hour for 24 h. In consultation with the neurosurgery service, supportive treatment and the following tests were requested to rule out potential thrombosis causes: C, S protein, antithrombin3, and factor V Leiden, all of which were normal. Owing to the patient’s fever, a polymerase chain reaction (PCR) test for SARS-COV-2 was requested, and the result was positive. Supportive care with anticonvulsants and heparin administration was continued. Following the patient’s relative stability, a lumbar puncture (LP) was performed, which revealed normal pressure, clear cerebrospinal fluid (CSF), and normal culture, analysis, and serology; however, the CSF PCR test was positive for SARS-COV-2.

Remdesivir was added to the patient’s treatments at a dose of 100 mg daily for 5 days, with no dose reductions. Oral vitamin C at a dose of 500 mg every 12 h, a single dose of intramuscular vitamin D at 300,000 units, and intramuscular vitamin A at 50,000 units were also administered according to the national protocol for COVID-19.

After 3 days, the patient was weaned off the ventilator, the seizure was under control, and the CT scan showed no signs of the progression of the thrombotic lesion. However, the patient developed paralysis of the left lower extremities and speech impairment; hence, physiotherapy was initiated for the patient.

After discharge from the PICU, the patient developed recurrent coughs and moderate fever, and a chest CT scan was performed since pneumonia was suspected. Mild bilateral ground glass opacities were detected in the periphery of both lungs on the CT scan image. In addition to supportive therapy for COVID-19, the patient was administered intravenous ceftriaxone 500 mg two times daily and azithromycin syrup 100 mg, 10 ml daily. Owing to the improvement of symptoms and negative blood culture, the patient’s antibiotic treatment was discontinued. The patient was discharged with a prescription for subcutaneous enoxaparin 15 mg twice daily for 2 months and levetiracetam 500 mg twice daily. Two weeks after discharge, the chemotherapy maintenance phase was resumed.

During 3-month post-discharge follow-ups, the patient could walk and speak properly with physiotherapy and speech therapy assistance. After a 10-month follow-up, the patient’s ability to speak and walk had fully recovered, and bone marrow aspiration revealed the complete remission phase.

## Discussion

In the current report, we have demonstrated a known case of patient with pre-B-cell ALL who experienced CVA following the COVID-19 infection.

Patients with ALL are a subset of the immunocompromised population, putting them at increased risk for complicated diseases such as COVID-19. Owing to the highly immunosuppressive treatment regimen and prolonged treatment duration, the risk of COVID-19-related complications and life-threatening diseases may be higher in these patients than in the general population [15, 20, 21]. Fung and Babik demonstrated that immunocompromised patients with COVID-19 may be at a higher risk for severe diseases and poor outcomes [22].

A Chinese cohort of confirmed patients with COVID-19 found that the fatality rate of COVID-19 in patients with cancer is estimated to be 5.6%. Also, the severity of COVID-19 in cancer is 3.5 times higher than in the general population [23]. Furthermore, it is stated that more than 80% of hematologic patients require hospitalization, with about 15% requiring intensive care unit admission [24, 25]. Dai *et al*. demonstrated that patients with hematologic cancers infected with COVID-19 had the highest severity and mortality rate (33%) compared with patients with other malignancies [26]. Several other studies confirm the greater mortality rate among patients infected with COVID with hematologic cancers compared with those with solid tumors [27–29]. Infection with COVID-19 in children with hematologic malignancies appears to carry a low mortality risk. However, children with hematologic cancer have a higher mortality risk than those with solid organ malignancies [30].

CVA is a frequent consequence of COVID-19 and hematologic cancers. In children with cancer, stroke is a significant consequence that increases morbidity and mortality and necessitates higher levels of critical care [31]. Noje *et al*. showed that children with leukemia and brain tumors have the highest risk for stroke [19]. Parasole *et al*. predicted a prevalence of 1.97% for stroke in children treated for ALL [32]. Strokes in patients with cancer can be hemorrhagic or ischemic [33]. During the first 6 months after a cancer diagnosis, the risk of a hemorrhagic stroke is greater than that of an ischemic stroke in patients with cancer, which is also true for patients with leukemia [34]. The mechanisms of stroke in patients with cancer are multifactorial and may be directly related to cancer, consequences of cancer such as infections and hypercoagulability, or therapeutic and diagnostic interventions [35]. Infection, particularly brain infection, is one of the important risk factors for stroke. Owing to anti-cancer therapy, patients with cancer are immunocompromised; therefore, infections can easily spread throughout the body. Sepsis and its combination with disseminated intravascular coagulation (DIC) can induce septic cerebral infarction [36]. COVID-19 has been one of the most widespread diseases worldwide over the past 2 years, and is known to raise the risk of stroke. The risk of ischemic stroke in COVID-19 is reported to be 5%. In COVID-19, hemorrhagic stroke is uncommon but has occurred in a few cases. The mechanisms of ischemic stroke in COVID‐19 are hypercoagulopathy, vasculitis, and cardiomyopathy. A hemorrhagic stroke in COVID-19 may result from the following probable causes: the inflammatory response, or cytokine storm, that occurs alongside the infection, and the angiotensin-converting enzyme 2 (ACE-2) receptors that are expressed in intracranial arteries, which permit the SARS-CoV-2 virus to damage the arteries and lead to rupture of the vessel wall [12, 37]. Another study revealed the following reasons underlying the occurrence of stroke in COVID-19: (1) Susceptibility of a group of patients to hypoxemia-induced cerebrovascular damage, (2) Sepsis-induced coagulopathy, which is widespread in patients with COVID-19 with high D-dimer and fibrinogen levels, and (3) ACE-2 receptors and cytokine storm. Cytokine storm is an adverse effect of immunity to COVID-19, defined by uncontrolled hyperactivation of the immune system. These excessive cytokines can cause plaque rupture and superimposed thrombosis [38].

Given that both the COVID-19 infection and underlying cancer can raise the patient’s risk of stroke, the presence of both conditions may raise the risk of stroke [39]. However, there are still some debates about the synergy between these two in thrombosis [40]. Owing to this controversy, additional research is required on these populations before a conclusion can be reached.

Owing to the rarity of this case, one of the limits of the disease’s management was the lack of a defined treatment protocol at the time of the patient’s treatment and the partial implementation of preventative measures at the beginning of the patient’s treatment. On the other hand, because of the presence of multiple factors in the occurrence of stroke in children with COVID-19 who have a history of leukemia, it is impossible to completely prevent this occurrence. However, this case provides useful information for the management of future patients.

## Conclusion

Owing to the association between COVID-19 and leukemia with hypercoagulopathy, as well as the susceptibility of children with leukemia to severe complications of COVID-19, children with leukemia and suspected COVID-19 must be hospitalized and given special care. Additionally, proper measures should be taken to prevent the formation of blood clots to reduce the risk of consequences such as stroke. Moreover, In the presence of stroke-suspicious symptoms, physicians should initiate essential evaluations as well as cardioembolism prevention or treatment.

## Data Availability

Not applicable.

## Abbreviations

COVID-19: Coronavirus disease 2019
SARS-CoV-2: Severe acute respiratory syndrome coronavirus
ANC: Absolute neutrophil count
CT: Computed tomography
PICU: Pediatric intensive care unit
CVA: Cerebrovascular accident
ACE-2: Angiotensin‐converting enzyme 2
ALL: Acute lymphoblastic leukemia
Pre-B-cell ALL: Precursor B-cell acute lymphoblastic leukemia
TdT: Terminal deoxynucleotidyl transferase
HLA-DR: Human Leukocyte Antigen-DR isotype
CyIgM: Cytoplasmic immunoglobulin M
CSF: Clear cerebrospinal fluid
ALT: Alanine aminotransferase
AST: Aspartate aminotransferase
ALP: Alkaline phosphatase
PCR: Polymerase chain reaction
DIC: Disseminated intravascular coagulation
